# Reply to Comment on “Jun, S.Y.; et al. Tumor Budding and Poorly Differentiated Clusters in Small Intestinal Adenocarcinoma” *Cancers* 2020, *12*, 2199

**DOI:** 10.3390/cancers12102987

**Published:** 2020-10-15

**Authors:** Sun-Young Jun, Seung-Mo Hong

**Affiliations:** 1 Department of Pathology, Incheon St. Mary’s Hospital, College of Medicine, The Catholic University of Korea, Seoul 21431, Korea; 2 Department of Pathology, Asan Medical Center, University of Ulsan College of Medicine, Seoul 05505, Korea

We thank Giuffrida et al. for their interest in our manuscript [1]. Based on their comment, we read the paper by Arpa et al., which analyzed tumor budding (TB) and poorly differentiated clusters (PDC) in 47 Crohn’s disease-associated, non-ampullary small intestinal adenocarcinomas (SIACs) [2]. They specifically concentrated on TB and PDC at the tumor invasive front (pTB and pPDC) and confirmed their own prognostic values predicting patient survival [2]. Furthermore, they proposed a combined pTB and pPDC score, which was called “a combined invasive front score”, to clarify low- and high-grade Crohn’s disease-associated SIACs [2]. However, we could not find this recent study by Arpa et al. [2] when we had performed a literature search of the electronic databases for our study. Therefore, we agree and accept the comment by Giuffrida et al. regarding the first study investigating the prognostic impact of pTB and pPDC in SIACs [1]. 

Our paper addressed the question of whether there is a difference in the prognostic significance of TB and PDC observed at the peritumoral invasive front and inside the tumor in SIACs. Compared to the study by Arpa et al., the distinct point of our study was a detailed comparative analysis of intra-tumoral TB and PDC (iTB and iPDC) as well as pTB and pPDC in a relatively large cohort of patients with SIAC (*n* = 236) [3]. We verified the inter-relationships, clinicopathological importance, and prognostic values of each variable of TB and PDC. Significant associations of pTB with pPDC and iTB were identified. TB and PDC, irrespective of peritumoral and intratumoral regions, showed similar aggressive behavior and adverse prognostic roles in SIACs. Despite independent assessment of the prognostic impact of each variable of TB and PDC by multivariate analysis, a significant predictive value was obtained only for pTB. Nevertheless, when the assessment of pTB and/or pPDC is difficult owing to the absence of the invasive front in the biopsy specimen, iTB and/or iPDC may help select the patients who require surgical resection or adjuvant chemotherapy and predict the tumor response and clinical outcome in patients undergoing chemotherapy, as previously described in studies of colorectal carcinomas [4,5,6]. However, further studies are needed to clarify the utility of iTB and/or iPDC in biopsy specimens for indicating the therapeutic choice, tumor response, and prognosis of patients with SIAC.
